# Synthesis and Biological Evaluation of a Novel Glycidyl Metharcylate/Phaytic Acid-Based on Bagasse Xylan Composite Derivative

**DOI:** 10.3390/polym13132084

**Published:** 2021-06-24

**Authors:** Mingkun Li, Heping Li, Hongli Liu, Zhiming Zou, Chaoyu Xie

**Affiliations:** College of Chemistry and Bioengineering, Guilin University of Technology, Guilin 541004, China; lmk19961231@gmail.com (M.L.); lhli06@163.com (H.L.); 357921449@163.com (Z.Z.); 1427460839@163.com (C.X.)

**Keywords:** bagasse xylan composite derivative, biocompatible composite material, esterification graft reaction, biological evaluation, molecular docking

## Abstract

The development of natural biomass materials with excellent properties is an attractive way to improve the application range of natural polysaccharides. Bagasse Xylan (BX) is a natural polysaccharide with various biological activities, such as antitumor, antioxidant, etc. Its physic-chemical and biological properties can be improved by functionalization. For this purpose, a novel glycidyl metharcylate/phytic acid based on a BX composite derivative was synthesized by a free radical polymerization technique with glycidyl metharcylate (GMA; ^GMA^BX) and further esterification with phytic acid (PA; ^GMA^BX-PA) in ionic liquid. The effects of the reaction conditions (i.e., temperature, time, initiator concentration, catalyst concentration, GMA concentration, PA concentration, mass of ionic liquid) on grafting rate(G), conversion rate(C) and degree of substitution(*DS*) are discussed. The structure of the composite material structure was confirmed by FTIR, ^1^H NMR and XRD. SEM confirmed the particle morphology of the composite derivative. The thermal stability of ^GMA^BX-PA was determined by TG-DTG. Molecular docking was further performed to study the combination mode of the ^GMA^BX-PA into the active site of two lung cancer proteins (5XNV, 2EB2) and a blood cancer protein (2M6N). In addition, tumor cell proliferation inhibition assays for BX, ^GMA^BX-PA were carried out using the 3-(4,5-dimethylthiazol-2-yl)-2,5-diphenyltetraz -olium bromide (MTT) method. The results showed that various reaction conditions exhibited favorable gradient curves, and that a maximum G of 56% for the graft copolymerization and a maximum *DS* of 0.267 can be achieved. The thermal stability was significantly improved, as demonstrated by the fact that there was still 60% residual at 800 °C. The molecular docking software generated satisfactory results with regard to the evaluated binding energy and combining sites. The inhibition ratio of ^GMA^BX-PA on NCI-H460 (lung cancer cells) reached 29.68% ± 4.45%, which is five times higher than that of BX. Therefore, the material was shown to be a potential candidate for biomedical applications as well as for use as a heat resistant material.

## 1. Introduction

Natural polysaccharides have been proposed as materials from which to produce functional materials and alternatives to petroleum-based materials. The use of polysaccha- rides has been proposed for drug carriers with slow release function [1,2,3], complex films with antibacterial properties [4,5], etc. Xylan, the second most widespread polysaccharide after cellulose, can be enhanced or given new capabilities by the introduction of new groups. The anticancer activity of xylan has been demonstrated; for example, xylan extracted from corn cobs by Cao et al. [6] was shown to possess biological activity against cervical and lung cancer. Some modification methods, such as grafting, esterification and etherification, have been reported, and their properties have been improved to a certain extent. Some grafted monomers, such as polyhexa-methylene guanidine hydrochloride [7] and acrylamide [8,9] have been due to their excellent antibacterial and mechanical properties. In esterification, acids such as sulfuric acid [10,11], stearic acid [12] and butyric acid [13] have been used as esterification agents to synthesize the functional derivatives of xylan, for instance, with anti-HSV, anticoagulation and anti-inflammatory properties. With the free radical polymerization technique, a polymeric chain was incorporated into the xylan backbone through a covalent chemical bond. These strategies extend the narrow processing window of bagasse xylan derivatives [14,15]. Nevertheless, it should be noted that conventional monografting and monoesterification have several limitations. Firstly, conventional monografting and monoesterification provide a single functionality due to the small variety of introduced groups; and secondly, the normal modified derivative demonstrates poor biological activity [16,17].

Dual modifications further improve the properties and uses of xylan [18]. Kong et al. [19] synthesized composite hydrogels from maleic anhydride-modified xylan under the action of ultraviolet radiation; the resulting composite hydrogels exhibited uniform porous structures and dual temperature/pH sensitivity. Simkovic et al. [20] found that films synthesized by the quaternization and sulfation of beech xylan had better mechanical properties than those synthesized by sulfation alone.

Glycidyl methacrylate (GMA) is a widely used vinyl monomer that has attracted a lot of attention in the medical and material fields due to its stable ternary ring structure [21,22]. For example, Roshanali et al. [23] reported the synthesis of a core-shell nanoparticle via grafting, which demonstrated enhanced physical stability. Phytic Acid (PA) containing phosphate groups can be bonded by esterification. Li et al. [24] synthesized PVA/PA polymer sponges by esterification of polyvinyl alcohol (PVA) and PA under acidic conditions and ultrasonic radiation, and demonstrated that the resulting PVA/PA polymer sponges were composed of PVA and PA linked by ether and phosphonic acid bonds, with thermal stability up to 416.5 °C and surface resistivity of up to 5.76 × 10^4^ ohm/square.

In light of above-mentioned research, a novel biocompatible composite derivative was synthesized with the aim of producing functional materials using the free radical polymerization technique and esterification. It was our hope to synthesize xylan derivatives with high levels of activity and stability. Meanwhile, the monomer GMA and the esterifying agent PA selected in this paper were not introduced into bagasse xylan at the same time. The BX was grafted with GMA to obtain a ^GMA^BX-grafted copolymer by using potassium persulfate as the initiator. Then, ^GMA^BX-PA was synthesized from ^GMA^BX with PA in the presence of ammonium persulfate as a catalyst and ionic liquid, used instead of an organic solvent. The aim of the present study was to evaluate the effects of dual modification (i.e., free radical polymerization and esterification) on the stability and biological properties of BX in order to determine whether biocompatible xylan derivatives can be used as active materials in wide range of applications.

## 2. Materials and Methods

In this chapter, the sources of experimental compounds, the extraction process of BX, the synthesis of ionic liquids ^GMA^BX, ^GMA^BX-PA, and the means of testing are introduced.

### 2.1. Materials

Glycidyl methacrylate (GMA; AR; 106-91-2), phytic acid (PA; AR; 83-86-3) and emulsifier OP-10 were acquired from Aladdin, ShangHai, China. Acrylated chlorine (AR; 107-05-1),1-methyl imidazole (AR; 616-47-7), absolute ethanol (AR; 64-17-5), potassium persulfate (AR; 7727-21-1), ammonium persulfate (AR; 7721-54-0) and acetone (AR; 67-64-1) were purchased from Kaitong Chemical Factory, TianJin, China. Sodium hydroxide (AR; 1310-73-2) and hydrochloric acid were purchased from Xilong Chemical, XiAn, China. All of the above solvents and reagents were used without further purification. Double distilled water was used as a solvent for the grafted copolymer.

### 2.2. Extraction of Bagasse Xylan

The extraction and purification of xylan from bagasse were performed following the alkali distillation method described in [25,26]. The bagasse was dried at 60 °C for 48 h and ground. Subsequently, the bagasse powder was washed with water for 3 h at 80 °C, and then dipped in NaOH(4%) for 24 h at 30 °C (solid-liquid ratio 1:10). The mixed liquid was pressed to obtain the extracting liquid, which was then adjusted to neutral pH with hydrochloric acid. Bagasse xylan was separated by settling after the addition of absolute ethanol. BX (precipitate) was separated by filtration, washed several times, and dried at 60 °C for 12 h.

### 2.3. Preparation of 1-Ally-3-methylimidazole Chloride

First, 1-methylimidazole (14.5 mL) and allyl chloride (20 mL) were placed in a round-bottom flask (250 mL) equipped with a condenser and magnetic stirrer. Afterwards, the equipment was placed under vacuum with a circulating water evacuation pump. The mixture was stirred at 25 °C for about 60 min, and then at 55 °C for about 8 h. Finally, the resulting crude product was vaporized with excess allyl chloride via a rotary evaporator. The prepared 1-allyl-3-methylimidazolium chloride (AmimCl) was stored in a refrigerator [27,28].

### 2.4. Graft Copolymerization Modification of Bagasse Xylan with Glycidyl Methacrylate (^GMA^BX)

BX was modified with GMA by a free radical polymerization technique [29,30]. In brief, BX (1.3 g) was scattered in double distilled water and transferred into a four-neck round-bottom flask, to which GMA (10 mL, 22%), OP-10 (0.5 mL, add OP-10 to disperse GMA in water), and potassium persulfate (10 mL, 5.26%) were added slowly. The temperature was increased to 82 °C, and the reaction was performed for 10 h under magnetic stirring and reflux.

The intermediate product (^GMA^BX) was transferred to a beaker containing 35 mL acetone. To solvent was removed by vacuum filtration, and ^GMA^BX was washed several times with ethanol and distilled water. Next, the ^GMA^BX was lyophilized under reduced pressure to obtain pure ^GMA^BX.

**Figure 1:**
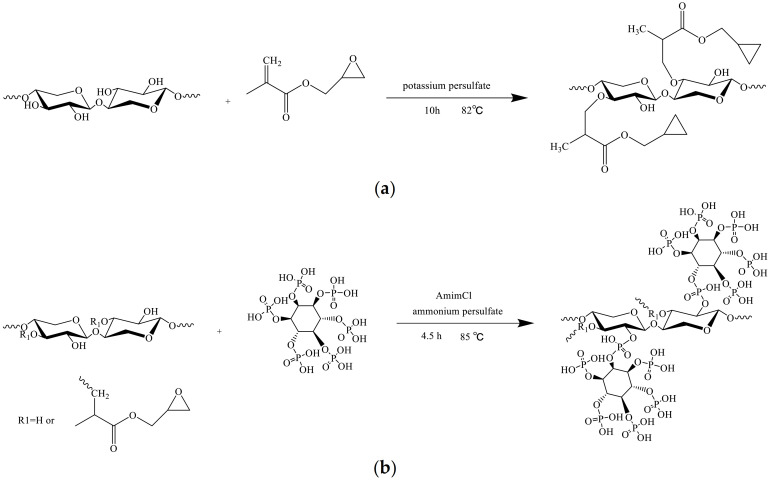
The synthesis routes of (**a**) ^GMA^BX and (**b**) ^GMA^BX-PA.

A schema for the chemical modification of BX with GMA is represented in Figure 1a.

### 2.5. Bagasse Xlyan Graft Copolymer Functionalization with Phytic Acid (^GMA^BX-PA)

The ^GMA^BX was functionalized with PA according to the method of esterification [31]. Initially, the AmimCl (a yellow liquid) was added into a four-neck round-bottom flask at 35 °C, Afterward, ^GMA^BX (1 g) and PA (2.8 mL) were added into AmimCl. Once dissolved, ammonium persulfate (0.2 g) was added. The temperature was increased to 85 °C, and the reaction was performed for 4.5 h under magnetic stirring and reflux.

The final material (^GMA^BX-PA) was added to a beaker containing 30 mL ethanol. After vacuum filtration, the ^GMA^BX-PA was washed with propanone and distilled water and lyophilized for 24 h at −35 °C.

A schema for the functionalization of ^GMA^BX with PA is represented in Figure 1b.

### 2.6. Determination of Grafting Rate and Monomer Conversion Rate

Impurities were removed from ^GMA^BX by soxhlet extraction with cyclohexane for 24 h. The grafting ratio (G) and conversion rate (C) of ^GMA^BX were calculated as follows [32]:

(1) $$G=\frac{W_{1}-W_{0}}{W_{0}}\times 100\%$$

(2) $$C=\frac{W_{1}-W_{0}}{W_{2}}\times 100\%$$

where *W*_0_ is the weight of BX, *W*_1_ is the weight of ^GMA^BX, and *W*_2_ is the weight of GMA.

### 2.7. Determination of Degree of Substitution

The degree of substitution (*DS*) of the product was determined with the acid-base titration method [33]. ^GMA^BX-PA (0.5 g) was placed into a 50 mL conical flask. Then, distilled water (10 mL) was added, and the mixture was shaken well. Subsequently, two drops of phenolphthalein indicator with a mass fraction of 5% were added and titrated to light red with NaOH standard solution at a concentration of 0.1 mol/L (fadeless within 30 s). Then, NaOH (2.5 mL) with a concentration of 0.5 mol/L was added to the conical flask and saponified by shaking at room temperature for 4 h. Finally, the mixture was titrated with 0.5 mol/L hydrochloric acid standard solution until it became colorless.

(3) $$DS=\frac{132\times w}{M-(M-1)\times w}$$

where *V*_0_ (mL) is the volume of HCl (0.5 mol/L) used to titrate the blank; *V*_1_ (mL) is the volume of HCl (0.5 mol/L) used to titrate the ^GMA^BX-PA; C_HCl_ is the concentration of the dilute hydrochloric acid; m (g) is weight of dried product; M (g/mol) is the molar of carboxylic acid; 132 (g/mol) is the molar mass of a xylose unit; and w (mol/g) is the mass of HCl consumed per gram of product.

### 2.8. Characterization

In this chapter, FTIR, XRD, TG-DTG, SEM, and 1H NMR were used as characterization tools, while molecular docking studies as well as the MTT method were used to explore the docking sites and antitumor activity. The subsections provide an explanation of the various methods and the scope of the tests.

#### 2.8.1. Fourier Transform Infrared (FTIR)

FTIR is a Nicolet-ISL0 from TA Instruments Co., LTD. (New Castle, Delaware, USA), with a spectral range of 400–4000 cm^−1^ and a resolution of 4 cm^−1^. BX, ^GMA^BX and ^GMA^BX-PA were analyzed using KBr tablets. Before the pressure sheet, the sample and KBr were dried at a constant temperature in a drying oven for 8 h. The sample and KBr were ground together at a ratio of 1:100–200 (*w*/*w*).

#### 2.8.2. Thermogravimetric Analysis (TG-DTG)

TG-DTG is an SDT-Q600 synchronous *TGA*/*DSC* analyzer produced by TA Instrument Co., LTD. (USA, New Castle, Delaware), the United States. The test range was adjusted to 35–800 °C and the heating rate was 10 °C/min to test the thermal decomposition of the xylan derivatives.

#### 2.8.3. X-ray Diffraction (XRD)

The XED patterns were collected for BX and ^GMA^BX-PA using a diffractometer (X ′PerT3 Powder XRD of Panaco Company, Almeo, The Netherlands). The scanning step length was 0.02626°, λ = 1.54056 A, the scanning speed was 0.6565°/s, and the scanning range was 5°–90°.

#### 2.8.4. Scanning Electron Microscope (SEM)

The morphology characteristics of ^GMA^BX-PA were determined in a JSM-6380LV SEM (JEOL Ltd., Akishima, Tokyo, Japan) operating at 10 KV acceleration voltage. Before the analysis, ^GMA^BX-PA were lyophilized and spuptter-coated with a thin layer of gold for 120 s.

#### 2.8.5. Hydrogen Nuclear Magnetic Resonance (^1^H NMR)

^1^H NMR spectra were collected using a AVANCE 500 MHz by Bruker BioSpin GmbH (Ettli Ngen, Germany). Deuterated dimethyl sulfoxide (CD_3_SO) was used as solvent for ^GMA^BX-PA. For that, from 0.8 to 1.0 mg of ^GMA^BX-PA was dissolved. The ^GMA^BX-PA was tested at a frequency of 500 MHz and a temperature of 298.2 K.

### 2.9. Molecular Docking

Molecular docking was carried out by computer. The structures of 5XNV, 2EB2, and 2M6N were obtained from Protein Data Bank (accessed on http://www.rcsb.org/pdb; accessed 3 April 2021) [34,35].

### 2.10. Tumor Cell Proliferation Inhibitory Assay

To explore the inhibitory effect of gmabx-pa on different cancer cells, NCI-H460 (human lung cancer cells), MGC80-3 (human stomach cancer cells), MDA-MB-231 (human breast cancer cells), and BEAS-2B (Bronchial Epithelium transformed with Ad12-SV40 2B) proliferation were assessed via MTT assay [36,37]. The test concentrations of the materials were 1, 10, 20, 50, and 100 μg/mL, respectively. The optical density (OD) was measured at 490 and 630 nm, with the former as a test and the latter as reference. Blank experiments only included culture fluid, MTT, and DMSO. Control experiments included cells, culture fluid, MTT, and DMSO. Sample experiments included materials, cells, culture fluid, MTT, and DMSO. All the experiments and measurements were done in triplicate, and arithmetic averages were taken during the data analysis and calculations. The results were analyzed statistically with Microsoft Office Excel 2010 and statistical package. The inhibition ratios of these materials to the cancer cells were calculated by the formula:

Relative Cell Proliferation Ratio (RCR%)

(4) $$\mathrm{Relative}\;\mathrm{Cell}\;\mathrm{Proliferation}\;\mathrm{Ratio}\;(\mathrm{RCR}\%)\;=\frac{\mathrm{OD}\left(\mathrm{sample},\;490\;\mathrm{nm}-630\;\mathrm{nm}\right)-\mathrm{OD}\left(\mathrm{blank},\;490\;\mathrm{nm}-630\;\mathrm{nm}\right)}{\mathrm{OD}\left(\mathrm{control},\;490\;\mathrm{nm}-630\;\mathrm{nm}\right)-\mathrm{OD}\left(\mathrm{blank},\;490\;\mathrm{nm}-630\;\mathrm{nm}\right)}\times 100\%\;\mathrm{Inhibition}\;\mathrm{ratio}=1-\mathrm{RCR}\%$$

In our tests, the standard deviation (SD) was estimated, and experiments were repeated to determine the experimental error. All data were reported as means ± standard deviation. The SD was calculated according to the following formula:

(5) $$\mathrm{SD}=\sqrt{\frac{\sum_{i=1}^{n}{\left(X_{i}-\bar{X}\right)}^{2}}{n-1}}$$

## 3. Results

### 3.1. Single Factor Analysis of Graft Copolymerization Reaction

**Figure 2:**
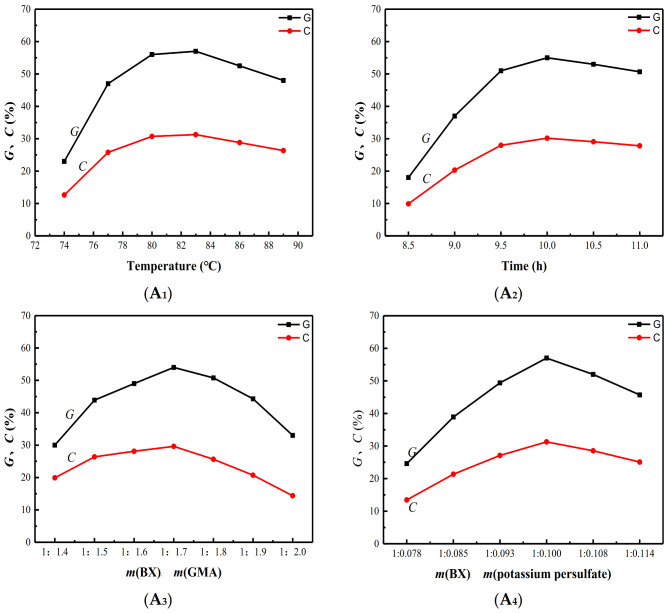
Effects of reaction conditions on G and C. (**A_1_**) The influence of reaction temperature on G and C. (**A_2_**) The influence of reaction time on G and C. (**A_3_**) The influence of mass of GMA on G and C. (**A_4_**): The influence of mass of potassium persulfate on G and C. The influences of A_1_, A_2_, A_3_ and A_4_ were discussed by keeping the other three factors constant.

Four variables factors were examined regarding graft copolymerization: reaction temperature (**A_1_**), reaction time (**A_2_**), mass ratio of potassium persulfate to BX (**A_3_**), and mass ratio of GMA to BX (**A_4_**). As shown in Figure 2, the influences of **A_1_**, **A_2_**, **A_3_**, and **A_4_** were evaluated by keeping the other three factors constant. The optimum reaction temperature was 82 °C, and the optimal grafting time was 10 h. According to the A3 and A4 curves, the optimal mass ratio of m(BX):m(GMA) with the addition of m(BX):m(potassium persulfate) was 1:1.7, and the optimal addition amount of m(BX):m(potassium persulfate) received by each of these curves was 1:0.1. Under optimal reaction conditions, the G and C of ^GMA^BX reached 56% and 29% respectively.

### 3.2. Single Factor Analysis of Esterification Reaction

**Figure 3:**
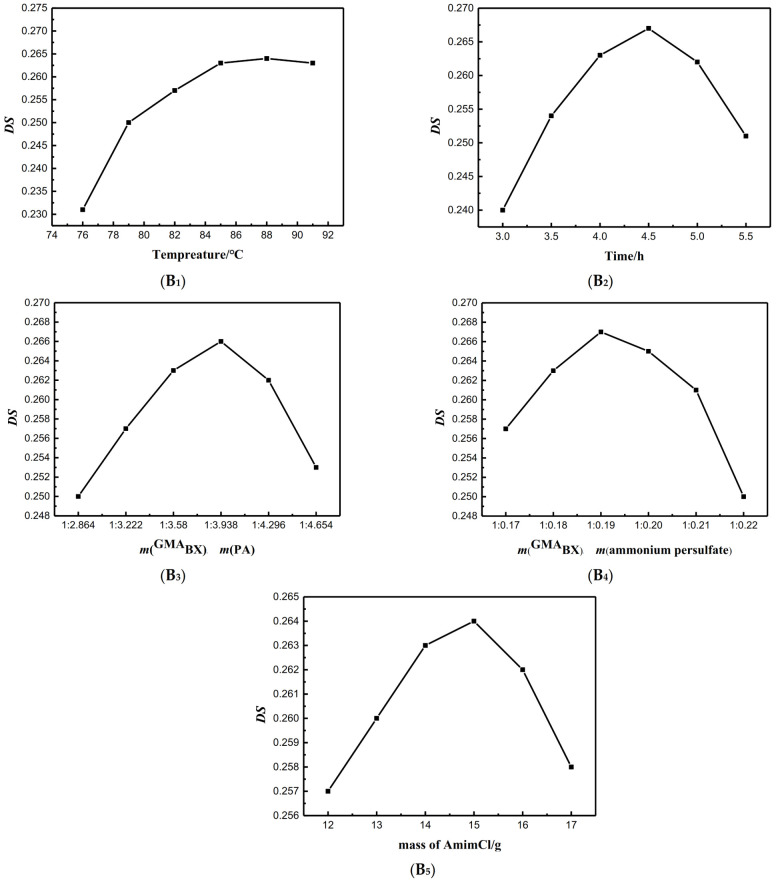
Effects of reaction conditions on *DS*. (**B_1_**) The influence of reaction temperature on *DS*. (**B_2_**): The influence of reaction time on *DS*. (**B_3_**): The influence of mass of PA on *DS*. (**B_4_**): The influence of mass of ammonium persulfate on *DS*. (**B_5_**): The influence of mass of AmimCl on *DS*. The influences of B_1_, B_2_, B_3_, B_4_ and B_5_ were examined by keeping the other four factors constant.

In order to optimize the esterification reaction, five variables were considered: esterification temperature (**B_1_**), esterification time (**B_2_**), mass ratios of ammonium persulfate to ^GMA^BX (**B_3_**), mass ratio of ^GMA^BX to PA (**B_4_**), and mass of AmimCl (**B_5_**). As shown in Figure 3, the influences of **B_1_**, **B_2_**, **B_3_**, **B_4_** and **B_5_** were evaluated by keeping the other four factors constant. For **B_1_** and **B_2_**, the optimal reaction temperature was 85 °C, and the optimal reaction time was 4.5 h. For **B_3_**, **B_4_** and **B_5_**, the optimal use ratio of m(^GMA^BX):m(ammonium persulfate) was 1:0.19, and the optimal addition amount of m(^GMA^BX):m(PA) was 1:3.938. The dosage of AmimCl was 15 g. Under the optimal reaction conditions, the *DS* of ^GMA^BX-PA reached 0.267.

### 3.3. Structure Analysis

Subsequent to the single-factor analysis to investigate the optimal reactions of the product synthesis, spectroscopic analyses and performance tests including FTIR, TG-DTG, XRD, SEM, ^1^H NMR were performed.

#### 3.3.1. FTIR Analysis of ^GMA^BX-PA

**Figure 4:**
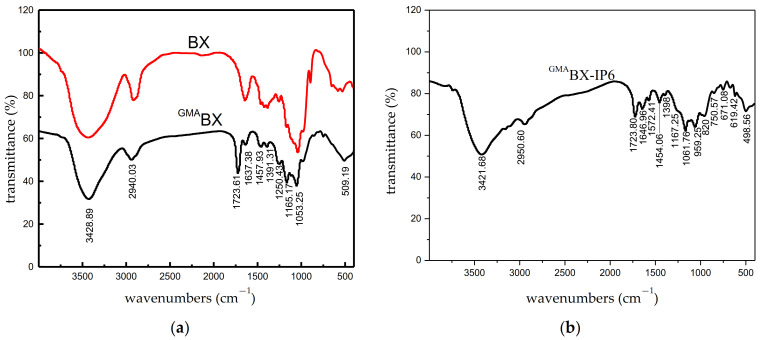
FTIR spectra of BX, ^GMA^BX and ^GMA^BX-PA. (**a**) FTIR spectra of BX and ^GMA^BX. (**b**) FTIR spectra of ^GMA^BX-PA.

Figure 4a shows the FTIR spectra for the graft-modified BX. The modification was confirmed by the presence and absence of characteristic bands from BX and GMA. For BX, stretching of –OH was observed at 3428.89 cm^−1^, and stretching of C–H, at 2940.03 cm^−1^, 1391.31 cm^−1^, and 1165.17 cm^−1^. The absence of the bands at 1053.25 cm^−1^ was characteristic of the asymmetrical stretching of the C–O–C epoxy-ring. The bands at 1723.61 cm^−1^ and 1250 cm^−1^ in the ^GMA^BX spectra confirmed the modification of BX with GMA. The bands were characteristic of the stretching of C=O and ternary ring vibrations from GMA. In Figure 4b, the bands at 1572.41 cm^−1^, 1061.76 cm^−1^, and 959.52 cm^−1^ were attributed to the P–O and P–O–P of PA. The new absorptions in the FTIR spectrum directly confirmed the conversion of BX into ^GMA^BX-PA.

#### 3.3.2. TG-DTG Analysis of ^GMA^BX-PA

**Figure 5:**
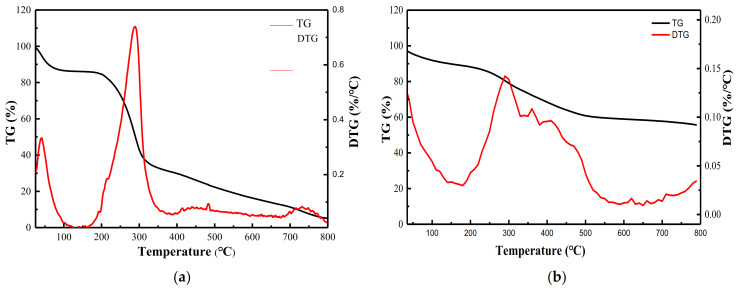
TG-DTG curve of BX and ^GMA^BX-PA. (**a**) TG-DTG curve of BX. (**b**) TG-DTG curve of ^GMA^BX-PA.

One of the important conditions for the thermal stability of polymers is the composition of their structures. The chemical modification of ^GMA^BX-PA led to a change in its thermal stability. Figure 5 shows the thermal properties of BX (**a**) and ^GMA^BX-PA (**b**). The thermal decomposition of BX can be divided into three stages, and the final product also exhibited weight loss in three stages. The rapid weight loss observed in the TG curve of BX in the early stage of heating, i.e., below 200 °C, was due to water loss. BX then lost 53% of its weight at 200–300 °C, mainly due to a loss of quality from the breakage and decomposition of glycosidic bondsand hydroxyl groups. BX was decomposed after 300 °C and lost 35% of its weight, mainly due to side-chain breakage. The sharp weight loss in the TG curve of ^GMA^BX-PA was due to water loss below 200 °C. ^GMA^BX-PA lost 27% of its weight at 200–500 °C due to breakages of graft branch chains. ^GMA^BX-PA lost 5% of its weight above 500 °C due to the decomposition of inositol hexaphosphate groups. Therefore, the ^GMA^BX-PA product had higher thermal stability than BX because of the GMA and PA groups introduced into the BX surface.

#### 3.3.3. XRD Analysis of ^GMA^BX-PA

**Figure 6:**
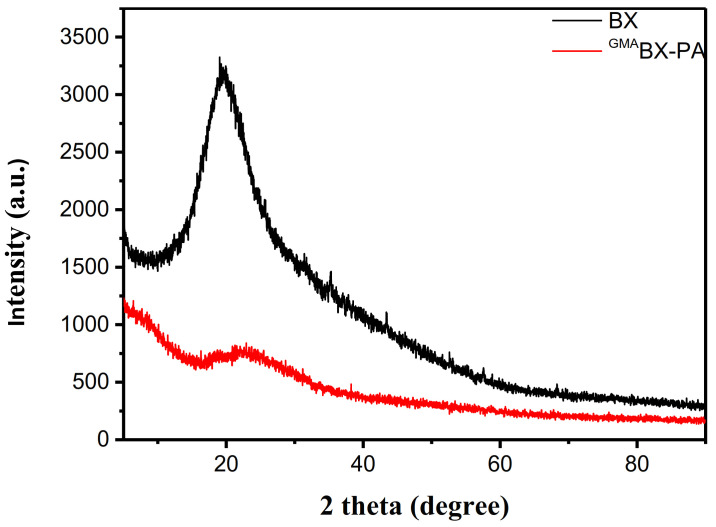
XRD pattern of BX and ^GMA^BX-PA.

The XRD patterns of BX and ^GMA^BX-PA are shown in Figure 6. As shown in Table 1, at diffraction angles of 10°, 16°, 20°, 23°, and 32°, the X-ray powder diffraction patterns of BX showed stronger diffraction peaks and weaker crystal morphology, indicating that BX had an amorphous structure. By a comparison with Table 2, it may be seen that ^GMA^BX-PA had new diffraction peaks at 6°, 7°, 28°, 38°, 47°, 51°, 68°, 75°, and 76°. This could be related to an increase in crystallinity due to the incorporation of GMA and PA into the backbone. The new characteristic peaks indicated successful grafting and esterification. It can be seen from Figure 6 that the X-ray powder graph of ^GMA^BX-PA in the range of 18–23° had a broad and blunt peak shape. The scattering angle at 20° was represented by anhydrous crystals, while the presence of an additional peak at 23° was attributed to the allomorphic tendon form of BX. The results indicated that new functional groups had been introduced, changing the structure of BX and possibly forming a new crystalline interfaces during grafting copolymerization and esterfication.

#### 3.3.4. SEM Analysis of ^GMA^BX-PA

**Figure 7:**
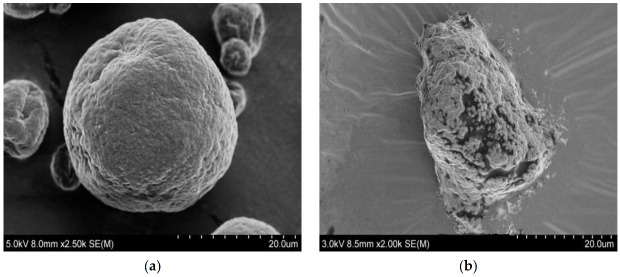

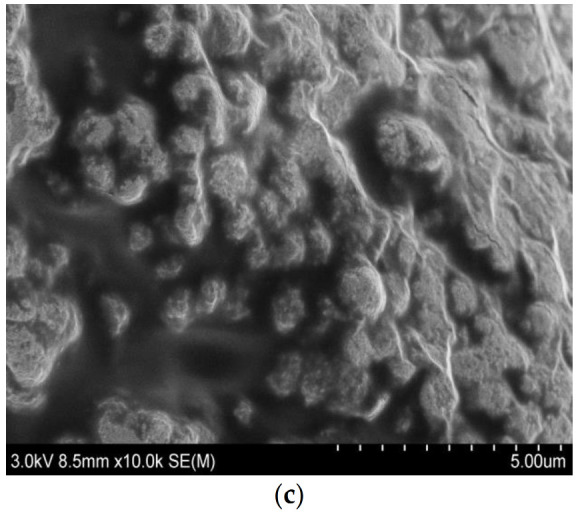
SEM images of BX and ^GMA^BX-PA. (**a**) SEM image of BX. (**b**) SEM image of ^GMA^BX-PA (**c**) SEM surface image of ^GMA^BX-PA.

Figure 7 shows microscopy images of BX and ^GMA^BX-PA. The synthetic method led changes in the morphology. The surface of BX (Figure 7a) was much coarser compared to that of ^GMA^BX-PA (Figure 7b). Many compact, heterogeneous particles were obtained on surface of the ^GMA^BX-PA due to the introduction of GMA and PA groups into the BX surface by graft and esterification.

#### 3.3.5. ^1^H NMR Analysis of ^GMA^BX-PA

**Figure 8:**
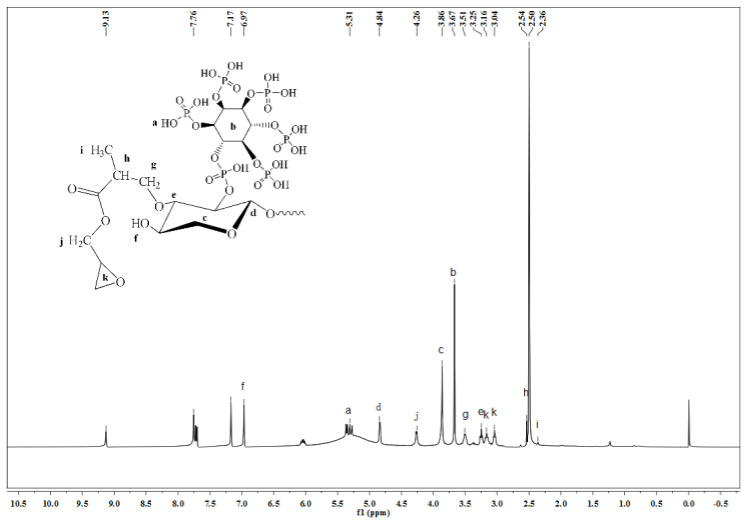
^1^H NMR spectrum of ^GMA^BX-PA.

The synthesized ^GMA^BX-PA was structurally characterized by ^1^H NMR. As shown in Figure 8, it showed a characteristic of DMSO at 2.50 ppm. The peaks at 3.25, 3.86, 4.84, and 6.97 ppm were hydrogen peaks in the BX structure. Peaks were observed at 5.31 ppm (P—OH), 3.67 ppm (hydrogen peak of benzene ring structure), 3.04 and 3.16 ppm (hydrogen peaks of the three-membered ring structure of GMA), 2.54 ppm (–CH–C=O), 4.26 ppm (–CH2–), and 2.36 ppm (–CH_3_). This tested the hypothesis that the ^GMA^BX-PA could be modified by grafting and esterification.

### 3.4. Molecular Docking Study

**Figure 9:**
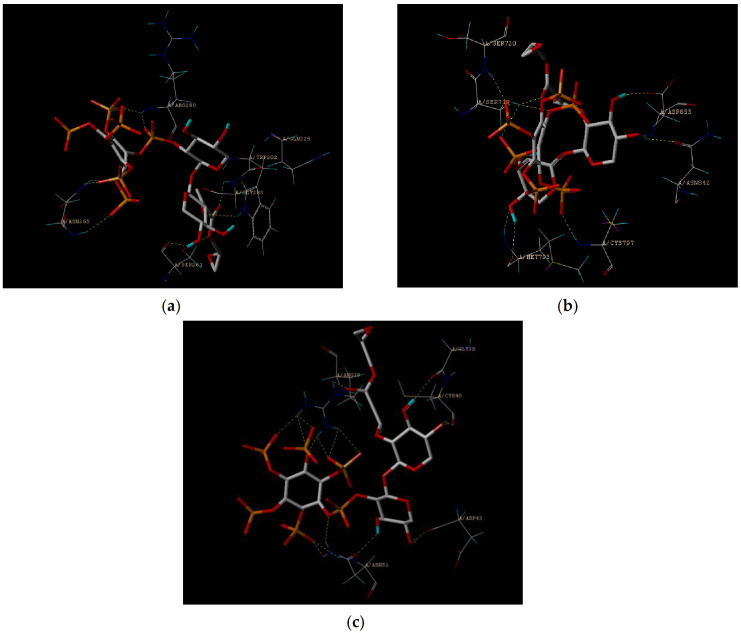
Images of the ^GMA^BX-PA link to the amino acid residue. (**a**) An image of the ^GMA^BX-PA link to the amino acid residue of 5XNV. (**b**) An image of the ^GMA^BX-PA link to the amino acid residue of 2EB2. (**c**) An image of the ^GMA^BX-PA link to the amino acid residue of 2M6N.

**Figure 10:**
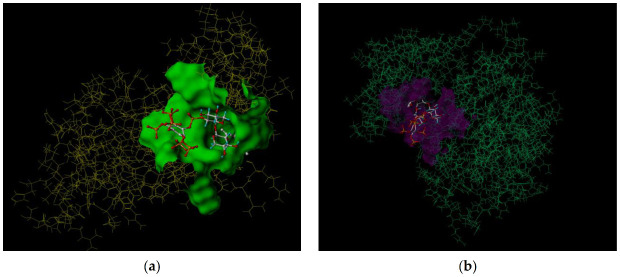

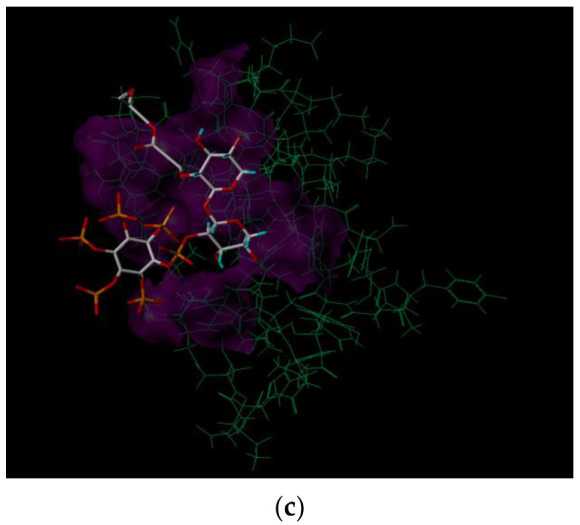
Images of ^GMA^BX-PA docking models. (**a**) An image of the ^GMA^BX-PA docking model of 5XNV. (**b**) An image of the ^GMA^BX-PA docking model of 2EB2. (**c**) An image of the ^GMA^BX-PA docking model of 2M6N.

The molecular docking method was used to simulate the docking of ^GMA^BX-PA with 5XNV, 2EB2, and 2M6N to determine the binding energy involved in the formation of the complex and the targeted inhibition of the molecular interactions with which it was formed. Figure 9 and Figure 10 show the docking sites and models of ^GMA^BX-PA with the three tumor proteins. We can see that ^GMA^BX-PA forms strong bonds with all three tumor proteins; therefore, it can be concluded that ^GMA^BX-PA has the ability to bind to cancer cells. The obtained scoring function (Table 3) showed that the docking effect of ^GMA^BX-PA was stronger in the cotton line than in the original BX. Therefore, it has been theoretically proven that ^GMA^BX-PA possesses strong antitumor activity.

### 3.5. Inhibition Analysis of Tumor Cell

The inhibition ratios of BX and ^GMA^BX-PA under different mass concentrations on cancer cell lines NCI-H460, MGC80-3, and MDA-MB-231, and human normal lung epithelial cells BEAS-2B, were evaluated. The results (tested by the key laboratory of pharmaceutical chemistry and drug molecular engineering at Guangxi Normal University) are show in Table 4.

The inhibition ratios of BX and ^GMA^BX-PA on human lung cancer cells (NCI-H460), human stomach cancer cells (MGC80-3), human breast cancer cells (MDA-MB-231), and human normal lung epithelial cells (BEAS-2B) were evaluated. The results (shown in Table 4) indicated that ^GMA^BX-PA has the potential to suppress 2.95 ± 1.13% of the growth of NCI-H460 cancer cells at concentrations equal or superior to 1 μg/mL. However, ^GMA^BX-PA has the potential to suppress 29.68 ± 4.45% of the growth of NCI-H460 cancer cells at high concentration (100 μg/mL). ^GMA^BX-PA can exert an inhibitory effect on the proliferation of the NCI-H460 cancer cells. Although the inhibitory ratio of ^GMA^BX-PA on MGC80-3 and MDA-MB-231 was not as good as that of NCI-H460, it still had strong inhibitory effects, i.e., 11.37 ± 3.29% on the growth of MGC80-3 cancer cells and 20.65 ± 3.82% on the growth of MDA-MB-231 cancer cells. Furthermore for normal cells, ^GMA^BX-PA showed low toxicity. The improved anticancer behavior of the products can be explained by their surface modifications with GMA and PA, which improved their stability and introduced active groups with anticancer properties.

## 4. Conclusions

In this work, ^GMA^BX copolymer was synthesized by a free radical polymerization technique, resulting in a G and C of 56% and 29%, respectively. The ^GMA^BX was further functionalized to prepare the ^GMA^BX-PA copolymer with the introduction of PA; *DS* was 0.297. The above results were confirmed by unifactor analysis. FTIR, ^1^H NMR, and XRD showed that epoxy groups and phosphate groups had been introduced into the BX. Surprisingly, the thermal stability was greatly improved, as demonstrated by TG-DTG and by the fact that there was still 60% residual at 800 °C. Molecular docking studies showed that the ^GMA^BX-PA had excellent docking activity with three tumor cell proteins, with strong binding energy and docking sites. Also, the MTT method demonstrated that the novel ^GMA^BX-PA inhibited lung cancer cells by 29.68 ± 4.45%, while being almost nontoxic to normal cells. This indicates that ^GMA^BX-PA has an excellent potential to treat tumor cells and biological materials for heat resistance. Moreover, the proposed synthesis method for ^GMA^BX-PA molecule could easily be streamlined, and therefore has commercial potential.

## Figures and Tables

**Table 1:** X-ray diffraction peaks of BX.

No.	Pos. [°2Th.]	FWHM [°2Th.]	Area [cts*°2Th.]	d-Spacing	Height [cts]	Rel.Int. [%]
1	10.2107	0.1692	360.3	7.89281	2129.5	47.9
2	16.4689	0.1033	138.1	7.09903	2005.05	45.11
3	20.4251	0.7768	2302.04	4.56975	4445.27	100
4	23.4479	0.0199	30.69	3.50022	2309.12	51.95
5	31.9634	0.5492	608.36	2.80004	1661.48	37.38

**Table 2:** X-ray diffraction peaks of ^GMA^BX-PA.

No.	Pos. [°2Th.]	FWHM [°2Th.]	Area [cts*°2Th.]	d-Spacing	Height [cts]	Rel.Int. [%]
1	6.4121	0.992	891.65	13.78465	1348.2	100
2	7.5506	0.9055	745.31	11.70863	1234.66	91.58
3	10.0881	0.304	233.69	8.76842	1153.2	85.54
4	16.4347	0.4085	190.36	5.39385	698.94	51.84
5	20.9515	0.4507	265.83	4.24013	884.81	65.63
6	23.1301	1.3628	1115.96	3.84544	818.87	60.74
7	28.658	0.5834	269.56	3.11503	693.04	51.41
8	31.1086	0.1764	64.96	2.875	552.27	40.96
9	38.1321	0.09	13.98	2.36007	233	17.28
10	47.2061	0.09	14.85	1.92543	165	12.24
11	51.8601	0.09	12.96	1.76305	144	10.68
12	68.0321	0.09	6.03	1.37809	67	4.97
13	75.9101	0.09	6.03	1.25347	67	4.97
14	76.2481	0.09	3.15	1.24875	35	2.6

**Table 3:** Molecular docking score.

		Total Score	D-Score	PMF-Score	*G*-Score	CHEM-Score	CScore	Global-Score
5XNV	BX	5.18	−182.58	−43.83	−235.78	−11.02	4	4
	^GMA^BX-PA	5.16	−685.39	−119.04	−357.38	14.14	5	5
2EB2	BX	8.60	−96.13	−108.38	−141.77	−17.66	4	4
	^GMA^BX-PA	8.89	−456.02	−179.09	−450.19	9.44	5	5
2M6N	BX	5.44	−105.00	−22.04	−136.06	−15.61	5	5
	^GMA^BX-PA	5.83	−453.63	−72.50	−268.03	−9.05	5	5

**Table 4:** The inhibition ratio of BX and ^GMA^BX-PA on different cancer cells and normal cells.

Sample	Mass Concentration/(μg/mL)	Inhibition Ratio/%			
		BEAS-2B	NCI-H460	MGC80-3	MDA-MB-231
BX	100	1.93 ± 0.48	4.62 ± 2.79	2.02 ± 0.57	3.16 ± 0.94
	50	1.72 ± 0.76	0.71 ± 0.22	0.24 ± 0.08	2.35 ± 0.72
	20	−0.26 ± 0.57	−0.24 ± 0.19	−0.15 ± 0.13	1.62 ± 0.47
	10	−2.94 ± 0.35	−2.97 ± 1.43	−2.99 ± 1.11	0.98 ± 0.33
	1	−5.61 ± 0.23	−4.33 ± 2.03	−3.27 ± 1.61	0.17 ± 0.12
^GMA^BX-PA	100	1.72 ± 0.59	29.68 ± 4.45	11.37 ± 3.29	20.65 ± 3.82
	50	0.96 ± 0.82	25.21 ± 4.56	7.39 ± 2.03	16.02 ± 3.71
	20	0.89 ± 0.77	17.02 ± 3.29	4.18 ± 1.37	13.29 ± 1.86
	10	−1.32 ± 0.39	9.77 ± 1.62	2.38 ± 0.24	10.41 ± 2.65
	1	−2.54 ± 0.43	2.95 ± 1.13	0.97 ± 0.28	7.16 ± 1.93

The inhibition ratio was calculated from 1—relative cell proliferation ratio. Relative cell proliferation ratio was calculated from (OD_sample_—OD_control_)∗100%.

## Data Availability

The date in this study presentedare avilable in this study. Additional information could be avilable on request from the corresponding author.
